# Neutrophil Adhesion and the Release of the Free Amino Acid Hydroxylysine

**DOI:** 10.3390/cells10030563

**Published:** 2021-03-05

**Authors:** Svetlana I. Galkina, Natalia V. Fedorova, Alexander L. Ksenofontov, Marina V. Serebryakova, Ekaterina A. Golenkina, Vladimir I. Stadnichuk, Ludmila A. Baratova, Galina F. Sud’ina

**Affiliations:** 1 A. N. Belozersky Institute of Physico-Chemical Biology, Lomonosov Moscow State University, 119991 Moscow, Russia; fedorova@genebee.msu.ru (N.V.F.); ksenofon@belozersky.msu.ru (A.L.K.); mserebr@mail.ru (M.V.S.); golyesha@mail.ru (E.A.G.); baratova@genebee.msu.ru (L.A.B.); sudina@genebee.msu.ru (G.F.S.); 2 Physical Department, Lomonosov Moscow State University, 119991 Moscow, Russia; stadn@polly.phys.msu.ru

**Keywords:** PMNLs (polymorphonuclear leukocytes), cell adhesion, cell secretion, hydroxylysine, procollagen-lysine, 2-oxoglutarate 5-dioxygenase 1-3 (PLOD 1-3), minoxidil, MMP-9, doxycycline, actin depolymerization, PI3K-Akt

## Abstract

During infection or certain metabolic disorders, neutrophils can escape from blood vessels, invade and attach to other tissues. The invasion and adhesion of neutrophils is accompanied and maintained by their own secretion. We have previously found that adhesion of neutrophils to fibronectin dramatically and selectively stimulates the release of the free amino acid hydroxylysine. The role of hydroxylysine and lysyl hydroxylase in neutrophil adhesion has not been studied, nor have the processes that control them. Using amino acid analysis, mass spectrometry and electron microscopy, we found that the lysyl hydroxylase inhibitor minoxidil, the matrix metalloproteinase inhibitor doxycycline, the PI3K/Akt pathway inhibitors wortmannin and the Akt1/2 inhibitor and drugs that affect the actin cytoskeleton significantly and selectively block the release of hydroxylysine and partially or completely suppress spreading of neutrophils. The actin cytoskeleton effectors and the Akt 1/2 inhibitor also increase the phenylalanine release. We hypothesize that hydroxylysine release upon adhesion is the result of the activation of lysyl hydroxylase in interaction with matrix metalloproteinase, the PI3K/Akt pathway and intact actin cytoskeleton, which play important roles in the recruitment of neutrophils into tissue through extracellular matrix remodeling.

## 1. Introduction

Neutrophils, normally circulating in the bloodstream, are able to penetrate the walls of blood vessels and invade infected tissues. In the focus of infection, neutrophils phagocytose and kill microbes with the help of special bactericidal agents that are secreted into the formed phagosomes, but also enter the extracellular environment. In the extracellular environment, aggressive products of neutrophil secretion can induce the development of inflammations that damage the surrounding tissues. In some metabolic disorders, recruitment of neutrophils into the tissue can occur even in the absence of infection. The release of aggressive bactericidal agents by recruited neutrophils causes the development of vascular and tissue pathologies during reperfusion after ischemia, myocardial infarction or diabetes [1,2,3]. The ability of neutrophils to penetrate into tissues can be compared with the ability of cancer cells to invade tissues and form metastases. To carry out invasion, tumor cells secrete enzymes that cause the necessary rearrangements in the extracellular matrix. Similar secretion, most likely, should also occur in neutrophils during migration and adhesion, but it is less studied. Understanding the biochemical processes that carry out the invasion of neutrophils can serve as the basis for the development of new means for the prevention of inflammatory processes caused by the penetration of neutrophils into tissue.

Previously, we found that the adhesion of neutrophils to fibronectin sharply stimulates the release of the free amino acid hydroxylysine into the extracellular environment, but does not affect the secretion of other amino acids by neutrophils [4]. Hydroxylysine is a metabolite of lysine produced by lysyl hydroxylase (LH or procollagen-lysine, 2-oxoglutarate 5-dioxygenase, PLOD). LH performs post-translational modification of collagen lysine residues in the rough endoplasmic reticulum (ER). A decrease in LH levels causes detrimental changes in collagen deposition and organization of the extracellular matrix, which, in turn, leads to changes in the morphology and cytoskeleton of the attached cells [5].

The role of hydroxylysine and LH in neutrophil physiology remains unknown. In contrast, the involvement of LH in tumor development and metastasis is being intensively studied. In tumor cells, LH is found not only intracellularly, but also in the extracellular space [6,7,8]. According to abundant evidence, secreted LH is a potent inducer of metastasis and a potential therapeutic target for cancer treatment. Three lysyl hydroxylases (LH1, LH2, LH3) encoded by PLOD1, PLOD2 and PLOD3 have been identified [9,10]. PLOD3 and PLOD2 are overexpressed and secreted by cells of lung cancer [7,11,12], glioma [13], glioblastoma [14], and pancreatic duct adenocarcinoma [15]. Increased expression of these enzymes clearly correlates with a poor prognosis in cancer patients. PLOD2 knockdown inhibited glioma cell proliferation, migration and invasion by inactivating PI3K/Akt signaling [16]. The PI3K/Akt pathway is often constitutively active in many types of cancer cells and is believed to promote cancer growth and metastasis [17,18,19].

In tumor cells, there is a strong positive correlation between PLOD3 expression and matrix metalloproteinases, which play a sufficient and necessary role in stimulating lung cancer metastasis. PLOD3 is thought to promote cancer metastasis by regulating STAT3, which in turn stimulates the expression of MMP-2 and MMP-9 [12]. Most MMPs are secreted, but some of their proteolytic activity is observed on the cell surface [20]. Recently, it was shown that the molecule responsible for the immobilization of MMP-9 on the cell surface of fibroblasts in the tumor stroma is LH3. Recruitment of MMP-9 to the cell surface triggers TGF-β activation and fibroblast differentiation into myofibroblasts, which enhance tumor progression through stroma remodeling [21]. MMP-9 can also activate TGF-ß in a functional complex with CD44 on the surface of keratinocytes and selected tumor cells [22]. CD44 is a hyaluronic acid receptor that plays a role in the organization of the extracellular matrix and in binding the extracellular matrix to the cytoskeleton [23]. Inhibition of actin polymerization by alendronate or cytochalasin D effectively blocks the formation of the CD44/MMP-9 complex, and also suppresses the secretion of the active form of MMP-9 and cell migration [24,25].

For neutrophils, it has been shown that the secretion of matrix metalloproteinases localized in tertiary granules plays an important role in cell migration and their recruitment into tissue [26]. The destruction of tight junction proteins by MMP-2 and MMP-9 is an important mechanism for the destruction of the blood–brain barrier, which promotes the penetration of leukemic cells into the central nervous system in acute leukemia [27]. Acute lung injury caused by *Streptococcus pneumoniae*, LPS or the highly toxic herbicide paraquat is characterized by neutrophil infiltration and extensive inflammation. Doxycycline, a tetracycline antibiotic, impairs neutrophil migration into the airspace of the lungs and reduces inflammation through a mechanism involving inhibition of the activity of MMP-9 derived from neutrophils [28,29,30]. Neutrophil-mediated release of MMP-9 induces acute inflammation during reperfusion after ischemia [31,32]. MMP-9 deficiency itself suppresses leukocyte recruitment and protects the liver from ischemia/reperfusion [33].

We would like to compare the biochemical processes that ensure the invasion and metastasis of tumor cells with the processes occurring during the recruitment of neutrophils into the tissues of the body. In this work, we studied the effect of inhibitors of LH (minoxidil), matrix metalloproteinase (doxycycline) and the PI3K/AKT pathway (wortmannin and AKT 1/2 inhibitor), as well as drugs that destroy the actin cytoskeleton integrity (cytochalasin D, latrunculin A, staurosporine, 4-bromophenacyl bromide and blebbistatin) on the morphology and secretion of proteins and free amino acids by neutrophils during adhesion to fibronectin using scanning electron microscopy, mass spectrometry and amino acid analysis.

## 2. Materials and Methods

### 2.1. Materials

Ficoll-Paque was obtained from Pharmacia (Uppsala, Sweden). Fibronectin was from Calbiochem (La Jolla, San Diego, CA, USA). Bicarbonate-free Hank’s solution, Ca2^+^-free Dulbecco PBS, cytochalasin D, minoxidil, doxycycline, wortmannin, Akt 1/2 inhibitor, cytochalasin D, latrunculin A, blebbistatin, staurosporine, 4-bromophenacyl bromide, and E64 were obtained from Sigma (Steinheim, Germany). Analytical chromatography conditions: eluent MCI Buffer L-8800-PH-1–4 and ninhydrin coloring solution kit for Hitachi 29,970,501 (Wako Chemicals, North Chesterfield, VA, USA). Coomassie Brilliant Blue G-250 was obtained from Serva, PMSF from MP Biomedical, trypan blue from Fluka AG, trypsin from Promega, glutaraldehyde from Ted Pella, carboxy-H_2_DCF-DA from Molecular Probe, (Eugene, OR, USA).

### 2.2. Neutrophil Isolation

Neutrophils were isolated from the blood of 22 healthy volunteers, who provided their informed consent, by experimental methods approved by the Bioethics Committee of Lomonosov Moscow State University (protocol number 69-o from 09.06.2016). Red blood cells were precipitated in the presence of 3% T-500 dextran at room temperature. Neutrophils were separated from plasma by centrifugation through Ficoll-Paque (density 1077 g/mL) in polypropylene tubes Costar (Corning, Monterrey, Mexico). The remaining erythrocytes were lysed in buffer (114 mM NH_4_Cl, 7.5 mM KHCO_3_, 100 µM EDTA), after which cells were washed twice in PBS. Neutrophils after washing before the experiment were stored in Dulbecco PBS containing 1 mg/mL glucose (without CaCl_2_).The purity of the neutrophilic fraction was 96–97%. To test viability of neutrophils after isolation or after the experiments cells were stained with trypan blue dye, which does not penetrate into viable cells, but stains dead cells. After collection of extracellular medium, neutrophils were incubated with 0.5 mM trypan blue in Hank’s solution for 15 min at 37 °C and washed. The number of dead cells (percent stained cells) was counted. 3000 cells were counted per group. The number of dead cells did not exceed 1% of the total number of cells. In order to exclude some activation of neutrophils during isolation, we keep the cells for 1.5 h in the dark at room temperature before the experiment.

### 2.3. Apoptosis Assessment

To detect phosphatidylserine externalization as an inherent part of cell death and to distinguish between apoptotic and necrotic changes, neutrophils were double-stained with Alexa Fluor-conjugated Annexin V (Annexin V-Alexa Fluor 488) and propidium iodide (PI; Merck, Darmstadt, Germany). Neutrophils were suspended at a density of 1 × 10^6^ cells/mL in HBSS supplemented with 20 mM HEPES, with or without the tested compounds, according to the experimental protocol. Cells were cultured in Eppendorf tubes for 30 min at 37 °C in a 5% CO_2_ incubator. After incubation, cells were collected by centrifugation at 270× *g* and washed once in cold PBS. Cell pellets were resuspended in HBSS/HEPES containing Annexin V-Alexa Fluor 488 commercial solution according to the manufacturer’s instructions. After 10 min on ice, PI solution (10 µg/mL HBSS/HEPES) was added for 5 min. The samples were analyzed on Cytoflex (Beckman Coulter, Europark Fichtenhain, Germany) using CytExpert 2.0 software. Fluorescence was detected by photomultipliers at 525 nm (Annexin V-Alexa Fluor 488) and 620 nm (PI). Hereinafter, leukocyte subpopulations were plotted as a dot plot and gated according to size and granularity. In total, 20,000 data events were collected for each acquisition.

### 2.4. Adhesion of Neutrophils to Fibronectin-Coated Substrata

Six-well culture plates Cellstar (Frichenhausen, Germany) were incubated for 2 h in Hank’s solution containing 5 μg/mL fibronectin at room temperature and washed. Neutrophils were plated to the protein-coated wells (3 × 10^6^ cells in 1.3 mL per well) in Hank’s solution containing 10 mM HEPES (HBSS/HEPES) (pH 7.35) for 20 min at 37 °C. Doxycyline (20 μM), minoxidil (200 μM), wortmannin (1 µM), Akt 1/2 inhibitor (25 µM), cytochalasin D (10 µg/mL), 4-bromophenacyl bromide (BPB) (25 µM), latrunculin A (1 µM), staurosporine (0.2 µM) and blebbistatin (10 µM) were added to the cells before plating. After the 20 min incubation, the extracellular medium was taken from the cells and inhibitors of metalloproteinase, serine and cysteine proteinases and myeloperoxidase (EDTA, 5 mM; PMSF, 200 µM; E64, 10 µM; and sodium azide, 0.025%, resp.) were immediately added to the samples. Unattached neutrophils were removed by centrifugation for 5 min at 400× *g* at room temperature. The extracellular medium samples were used to determine the amino acid and protein composition of neutrophil secretion. To determine amino acid composition, samples of extracellular medium collected from three similar wells were combined. To determine protein content, samples from six similar wells were combined.

### 2.5. Extraction from the Extracellular Medium and Concentration of Proteins

Proteins from the collected samples of the extracellular medium were extracted with an equal volume of a chloroform-methanol mixture (2:1, *v*/*v*). The mixture was vortexed and stirred in a shaker at 4 °C for 30 min. As was revealed previously, the chloroform phase contained almost all of the proteins detected in the samples of extracellular medium of neutrophils, while the water-methanol fractions contained only trace amounts of proteins. The chloroform phases were separated by 20 min centrifugation at 11,000× *g*, were collected and after evaporation of the solvent were subjected to electrophoresis.

### 2.6. Sodium Dodecyl Sulfate Polyacrylamide Gel Electrophoresis

For protein separation, we used one-dimensional sodium dodecyl sulfate electrophoresis under nonreducing conditions on a 15% polyacrylamide gel in the Mini-PROTEAN 3 Cell (Bio-Rad, Berkeley, CA, USA). Before electrophoresis, aliquots of the samples were boiled for 3 min in lysis buffer (Tris-HCl 30 mM, pH 6.8; SDS 1%; urea 3 M; glycerin 10%; bromophenol blue 0.02%). Separated proteins were stained with Coomassie Brilliant Blue G-250 0.22% (Serva).

### 2.7. Mass Spectrometry Identification of Proteins

MALDI-MS analysis was performed on MALDI-ToF-ToF mass spectrometer Ultraflextreme (Bruker, Karlsruhe, Germany) as previously described [4]. Proteins were separated by electrophoresis. Gel pieces were excised from each protein band, washed, dehydrated, and subjected to in-gel digestion with trypsin. The resulting peptides were extracted with 0.5% trifluoroacetic acid. Aliquots of samples were mixed on a steel target with 2.5-dihydroxybenzoic acid (30 mg/mL in 30% acetonitrile, 0.5% trifluoroacetic acid) and subjected to mass spectrometry analysis. The [MH]^+^ molecular ions were measured in reflector mode. The accuracy of monoisotopic mass peak measurement was within 30 ppm. Peptide fingerprint search was performed with the Mascot software 2.5.01 (http://www.matrixscience.com, accessed on 3 January 2021), Swissprot database through the mammalian proteins. Protein matches were considered significant (*p* < 0.05) if the score was >68.

### 2.8. Preparation of Samples for Amino Acid Analysis

Extracellular medium samples from three similar wells were collected, combined and concentrated with Centrivap Concentrator Labconco (Kansas City, MO, USA). The proteins were precipitated with sulfosalicylic acid (4.4%) and removed by centrifugation for 30 min at 18,000× *g*. The supernatants were collected and centrifuged through Vivaspin 500 membrane ultrafilters Membrane 3000 PES MWCO (Sartorius, Goettingen, Germany).

### 2.9. Amino Acid Analysis of Samples

Amino acids were quantified as described in [4], using L-8800 amino acid analyzer (Hitachi, Tokyo, Japan) in the standard mode according to the manufacturer’s user manual (Hitachi High-Technologies Corporation, Japan, 1998). Briefly, the samples were subjected to an ion-exchange column 2622SC (PH) (Hitachi, Ltd., P/N 855-3508, 4.6 × 80 mm^2^), eluted by step gradient of four sodium-acetate buffers at a flow rate of 0.4 mL/min at 57 °C. Colored products were detected by absorption at 570 nm. Data were processed using MultiChrom for Windows software (Ampersand Ltd., Moscow, Russia).

### 2.10. Reactive Oxygen Species (ROS) Formation Assay

Intracellular ROS (reactive oxygen species) formation was monitored by measuring green fluorescence of dichlorodihydrofluorescein diacetate (H_2_DCF-DA) oxidation product (DCF). The preliminary PMNL (polymorphonuclear leukocytes) labeling with H_2_DCF-DA was carried out in accordance with the manufacturer’s protocol. Briefly, human neutrophils were incubated with 5 µM carboxy-H_2_DCF-DA (Molecular Probe) for 60 min at room temperature followed by washing with PBS. Cells were then seeded in fibronectin-coated 96-well plates (1 × 10^6^/mL HBSS/HEPES) and incubated according to the experimental protocol at 37 °C in 5% CO_2_. Changes in fluorescence intensity upon excitation of 485 nm and emission of 538 nm were monitored for at least 30 min after the addition of inhibitors. Measurements were performed on a ClarioStar fluorescence microplate reader (BMG Labtech, Cary, NC, USA).

### 2.11. Scanning Electron Microscopy

The cover slips were incubated in a buffer containing 5 μg/mL fibronectin for 2 h at room temperature and washed. Neutrophils were plated to the fibronectin-coated cover slip (3 × 10^6^ cells in 2 mL per well) for 20 min incubation in a Hanks solution containing 10 mM HEPES (pH 7.35) at 37 °C. Doxycyline (20 µM), minoxidil (200 µM), wortmannin (1 µM), Akt 1/2 inhibitor (25 µM), cytochalasin D (10 µg/mL), 4-bromophenacyl bromide (BPB) (25 µM), latrunculin A (1 µM), staurosporine (0.2 µM) and blebbistatin (10 µM) were added to the cells before plating. After incubation, neutrophils attached to the cover slips were fixed in 2.5% glutaraldehyde in Hanks buffer without Ca^2+^ or Mg^2+^ ions, but containing 5 mM EDTA and 0.5 mM phenylmethylsulfonyl fluoride (PMSF), metalloproteinase and serine proteases inhibitors and 10 mM HEPES at pH 7.3. The cells were additionally fixed with a 1% solution of osmium tetroxide in 0.1 M sodium cacodylate containing 0.1 M sucrose at pH 7.3. After this, the cells were dehydrated in a series of acetones (10–100%) and dried in a Balzer apparatus at a critical point with liquid CO_2_ as the transition liquid. The samples were coated with gold/palladium sputter and examined at 15 KV using a Scanning Electron Microscope Camscan S-2. The area of attached neutrophils in the images obtained by scanning electron microscopy was determined quantitatively using ImageJ-win64 software.

### 2.12. Statistics 

The blood of 22 healthy donors was used in the work. Each experiment to determine the amino acid and protein composition of neutrophil secretion or neutrophil morphology was carried out at least 3 times using blood from different donors. Results are reported as mean ± SEM. Analysis of the statistical significance was evaluated using a two-way ANOVA with a Tukey’s multiple comparisons test using GraphPadPrism7 software. *—*p* ≤ values of less than 0.05 were considered significant; **—*p* ≤ 0.012; ****—*p* ≤ 0.0001.

## 3. Results

### 3.1. Effect of Minoxidil and Doxycycline on Neutrophil Morphology and Free Amino Acid Secretion during Adhesion to Fibronectin

We compared the effects of minoxidil and doxycycline on neutrophil morphology and free amino acid release during adhesion. Minoxidil is widely used as an LH inhibitor, but the mechanism of the inhibitory action remains to be seen. Minoxidil reduces the level of not only the protein lysyl hydroxylase, but also the relative mRNA, which indicates the transcriptional mechanism of inhibition [34]. It also acts as a vasodilator by opening the ATP-sensitive K^+^ channel [35,36]. Doxycycline, a tetracycline antibiotic, is widely used to inhibit matrix metalloproteinases at subantimicrobial doses. Inhibition is achieved by direct binding to Ca^2+^ or Zn^2+^ ions in the active site, inhibiting transcription, and indirectly by modulating endogenous inhibitors or activators [31,37,38].

**Figure 1:**
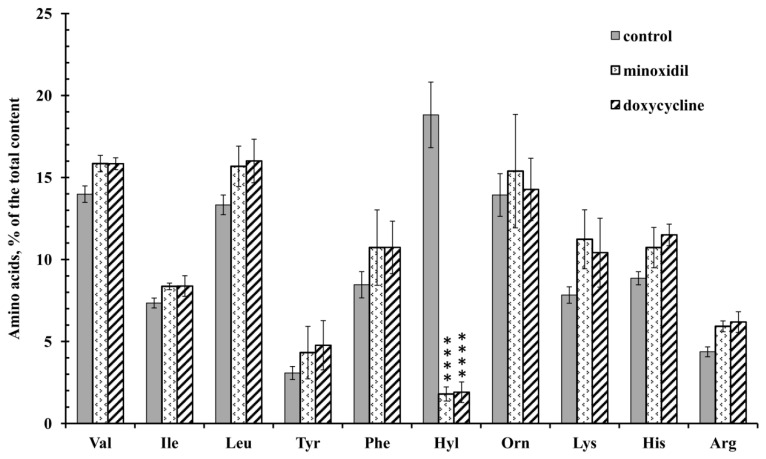
Free amino acid composition of secretion of the neutrophils during adhesion to fibronectin in the presence of minoxidil and doxycycline. Human neutrophils were incubated over fibronectin-coated substrata for 20 min under control conditions or in the presence of 200 µM minoxidil or 20 µM doxycycline. The amount of amino acid is represented as a percentage of the total content of the detected free amino acids (mean ± SEM). Amino acid profiles were obtained by summing the results of three independent experiments. ****—significant differences when compared to the value for the same amino acid in the control cells (*p* < 0.0001). (Val—valine; Ile—isoleucine; Leu—leucine; Tyr—tyrosine; Phe—phenylalanine; Hyl—hydroxylysine; Orn—ornithine; Lys—lysine; His-histidine; Arg—arginine).

Amino acid analysis showed that minoxidil dramatically reduced the release of hydroxylysine by neutrophils during adhesion to fibronectin (Figure 1). The effect was selective for hydroxylysine because minoxidil did not affect the release of other amino acids. A similar dramatic and selective inhibitory effect on the release of hydroxylysine was observed when neutrophils attached to fibronectin in the presence of doxycycline.

**Figure 2:**
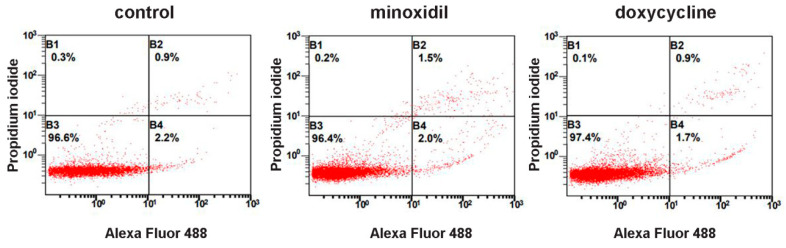
Neither minoxidil nor doxycycline is toxic to neutrophils under short-term exposure. Human neutrophils were cultured without additives or in the presence of 200 µM minoxidil or 20 µM doxycycline for 30 min. Immediately after that cells were stained with Annexin V, conjugated with Alexa Fluor 488 and propidium iodide, followed by flow cytometry analysis. Representative dot plots with proportions of viable (region B3), early apoptotic (region B4) and late apoptotic and necrotic cells (regions B2 and B1) are shown.

To exclude the possibility of the appearance of free amino acids in the extracellular medium as a result of cell destruction, we stained neutrophils after collecting the extracellular medium with trypan blue. The percentage of stained (dead) cells was less than 1% for control cells or cells treated with minoxidil and doxycycline. We also evaluated the externalization of phosphatidylserine and membrane integrity of neutrophils exposed to minoxidil or doxycycline for 30 min at 37 °C using Alexa Fluor conjugated annexin V/propidium iodide double-labeling, followed by flow cytometry. The data obtained for both inhibitors indicate the absence of toxic or destructive effects under the conditions of our experiment. (Figure 2).

**Figure 3:**
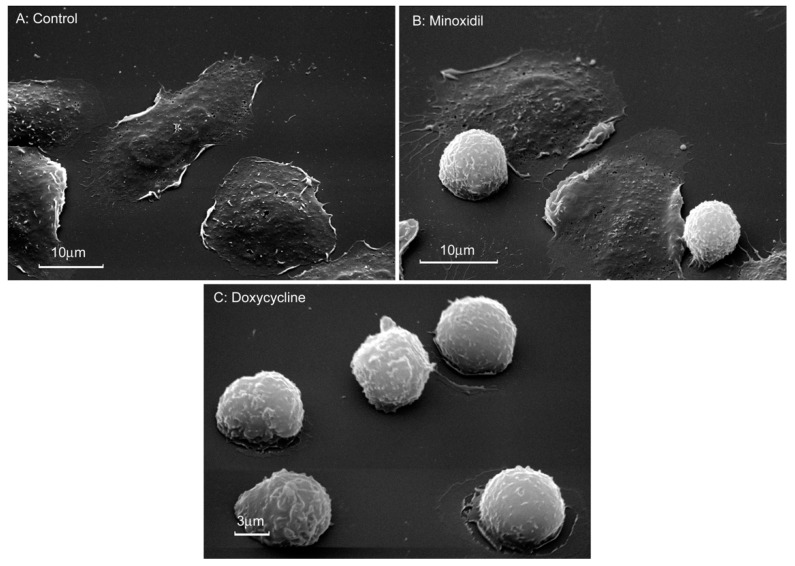
Morphology of neutrophils that were attached to fibronectin in the presence of minoxidil and doxycycline. Scanning electron microscopy images of human neutrophils that were attached to fibronectin-coated substrata for 20 min under control conditions (**A**) or in the presence of 200 μM minoxidil (**B**) or 20 µM doxycycline (**C**). Pictures represent typical images observed in three independent experiments.

Scanning electron microscopy showed that control neutrophils adhere and spread over the solid substrate coated with fibronectin (Figure 3A). In the presence of minoxidil, spreading was partially disturbed. The specimen contained spread and spheroid cells (Figure 3B). In the presence of doxycycline, neutrophil spreading was inhibited and all cells were spheroidal (Figure 3C).

Quantitative measurement of the area of attached neutrophils using the ImageJ program showed that the area of neutrophils attached to fibronectin in the presence of minoxidil or doxycycline was two or more times smaller than the area of control cells (Table 1). The significant standard error of the mean in experiments with minoxidil reflects large variations in cell spreading compared to cells treated with doxycycline, which have a more uniform, non-spread shape. These data confirmed that the inhibition of the release of hydroxylysine coincides with a partial or complete inhibition of neutrophil spreading.

### 3.2. Influence of Minoxidil and Doxycycline on the Formation of Intracellular Reactive Oxygen Species (ROS) during the Adhesion of Neutrophils to Fibronectin-Coated Substrates

**Figure 4:**
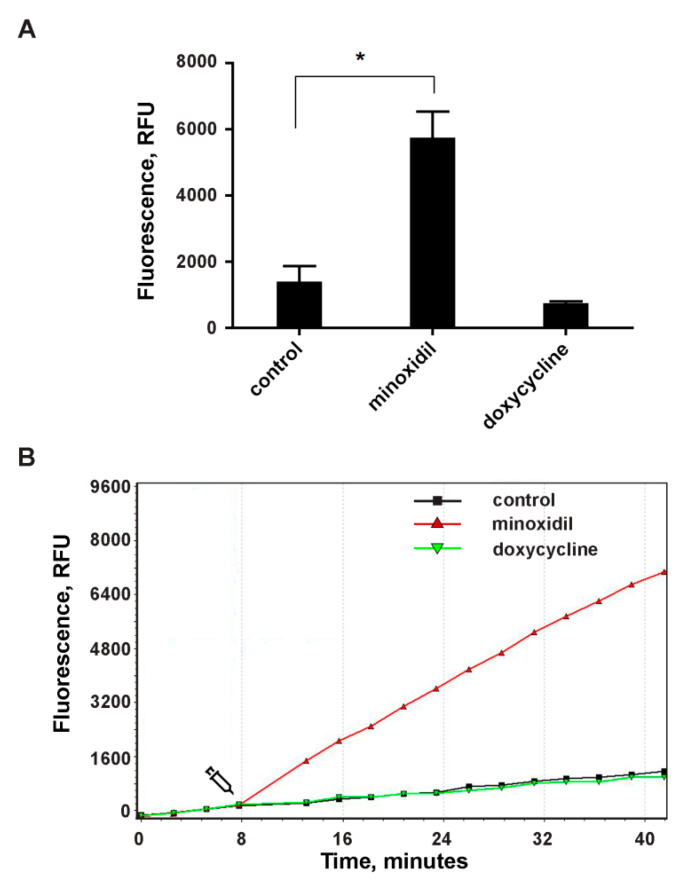
The effect of minoxidil and doxycycline on ROS formation during the adhesion of neutrophils to the fibronectin-coated substrate. H_2_DCF-DA-labelled neutrophils were cultured in fibronectin-coated flat bottom plates without additives or in the presence of 200 µM minoxidil or 20 µM doxycycline at 37 °C, 5% CO_2_. DCF (diacetate (H_2_DCF-DA) oxidation product) fluorescence signals (ex. 485 nm, em. 538 nm) were monitored with 2 min intervals. (**A**) Values represent the means ± SEM of fluorescence intensities (relative units) 30 min after the addition of inhibitors from three independent experiments. *—*p* < 0.05 (one-way ANOVA followed by Tukey’s multiple comparison test). (**B**) Representative DCF fluorescence kinetic curves for control (square, black), minoxidil (triangle, red) or doxycycline (inverted triangle, green) treated neutrophils.

We compared the effect of minoxidil and doxycycline on the formation of intracellular ROS during the adhesion of neutrophils to fibronectin. Adhesion of neutrophils to fibronectin is mediated by receptors β-1 and β-2 of the integrin family. Binding of β-2 integrin itself, in the absence of PMNL agonists, is capable of generating signals that trigger neutrophil spreading, ROS formation and chloride efflux [39]. Specific binding of β-1 integrin to a high-affinity binding site on fibronectin can lead to the assembly and activation of focal adhesion complexes and, in turn, to the initiation of a signaling cascade leading to the assembly of NADPH oxidase [40]. Our data showed that minoxidil and doxycycline, which demonstrated similar effects on neutrophil morphology and the hydroxylysine release, showed different effects on the ROS formation during neutrophil adhesion. Minoxidil stimulates, while doxycycline has practically no effect on ROS production (Figure 4). These data once again indicate the specific nature of the interaction of minoxidil and doxycycline in the processes of neutrophil adhesion and hydroxylysine secretion.

### 3.3. Inhibition of the PI3K/Akt Signaling Pathway Blocked Adhesion-Induced Secretion of Hydroxylysine by Neutrophils

The PI3K/Akt pathway is activated by a number of extracellular signals such as hormones, growth factors, and extracellular matrix components [41]. Activated PI3K (phosphatidylinositol-3-kinase) generates phosphatidylinositol-3,4,5-triphosphate, a lipid secondary messenger required for the translocation of serine/threonine protein kinase Akt (protein kinase B) into the plasma membrane, where it is phosphorylated. Activated Akt phosphorylates and regulates the function of many cellular proteins, including proteins of the cellular cytoskeleton and glucose metabolism, as well as endothelial nitric oxide synthase [42].

We studied the effect of PI3K and Akt inhibitors on neutrophil morphology and the release of free amino acids upon adhesion. We used wortmannin, a cell-permeable steroid metabolite of the fungus Penicillium funiculosum, which acts as a potent and irreversible PI3K inhibitor [43]. To inhibit Akt, we used a dual inhibitor of isoforms Akt 1 and 2 (Akt 1/2) with potential antitumor activity [44,45].

**Figure 5:**
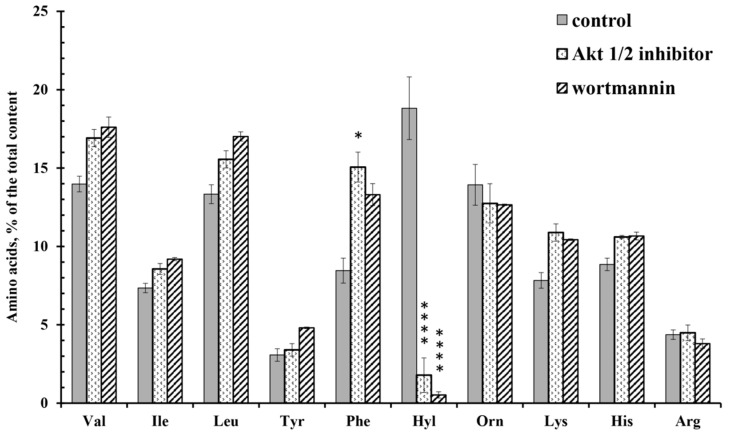
Free amino acid composition of secretion of the neutrophils during adhesion to fibronectin in the presence of inhibitors of PI3K/Akt pathway. Human neutrophils were incubated over fibronectin-coated substrates for 20 min in control conditions or in the presence of 25 µM Akt 1/2 inhibitor or 1 µM wortmannin. The amount of amino acid is represented as a percentage of the total content of the detected free amino acids (mean ± SEM). Amino acid profiles were obtained by summing the results of three independent experiments. *—significant differences when compared to the value for the same amino acid in the control cells (*—*p* < 0.01; ****—*p* < 0.0001). (Val—valine; Ile—isoleucine; Leu—leucine; Tyr—tyrosine; Phe—phenylalanine; Hyl—hydroxylysine; Orn—ornithine; Lys-lysine; His-histidine; Arg—arginine).

Wortmannin and the Akt 1/2 inhibitor sharply and selectively reduced the content of hydroxylysine, but did not significantly affect the content of other amino acids. In addition, the Akt 1/2 inhibitor significantly increased the secretion of phenylalanine, while wortmannin did not (Figure 5). The release of phenylalanine appears to be associated with the activation of Akt kinase via an alternative metabolic pathway. Other tyrosine or serine/threonine kinases can directly activate Akt in response to inflammation or growth factors. Hyperactivation of these alternative kinases is often associated with malignant neoplasms [46].

**Figure 6:**
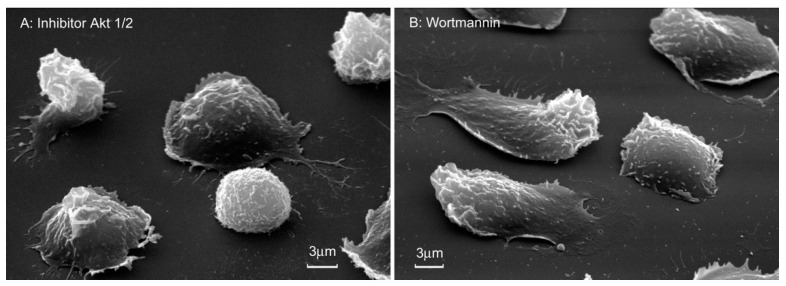
Morphology of neutrophils attached to fibronectin in the presence of inhibitors of the PI3K/Akt pathway. Scanning electron microscopy images of human neutrophils that were attached to fibronectin coated substrates for 20 min in the presence of 25 μM Akt 1/2 inhibitor (**A**) or 1 µM wortmannin (**B**). Images are typical images observed in three independent experiments.

Scanning electron microscopy showed that wortmannin and the Akt 1/2 inhibitor partially inhibited the spreading of neutrophils on fibronectin (Figure 6). Cell area measurements showed that wortmannin and an Act 1/2 inhibitor reduced the area of neutrophils attached to fibronectin in their presence by 3-fold (Table 1).

### 3.4. Drugs That Destroy the Integrity of the Actin Cytoskeleton Suppress the Release of Hydroxylysine and Stimulate the Secretion of Phenylalanine by Neutrophils during Adhesion to Fibronectin

We studied the morphology and composition of free amino acids on the secretion of neutrophils, which adhere to fibronectin in the presence of drugs that violate the integrity of the actin cytoskeleton. Cytochalasin D inhibits actin polymerization by binding to the pointed, rapidly growing ends of actin filaments. Latrunculin A binds to monomeric G-actin in a 1:1 ratio and prevents actin polymerization [47]. Staurosporine, a natural alkaloid of *Streptomyces staurosporeus*, has a high affinity for the ATP-binding site of protein kinases. Staurosporine can initiate depolymerization of the actin cytoskeleton in neutrophils, in particular, by inhibiting serine-3-cofilin neutrophil kinase [48]. The alkylating agent 4-bromophenacyl bromide (BPB) can act on the actin cytoskeleton via the leukocyte-specific actin-binding protein L-plastin [49]. Blebbistatin inhibits the activity of myosin-ATPase and, therefore, the actomyosin-based motility. This type of inhibition stabilizes the super-relaxed state of myofilaments, when the myosin heads are helical and interact with each other, but not with actin [50].

**Figure 7:**
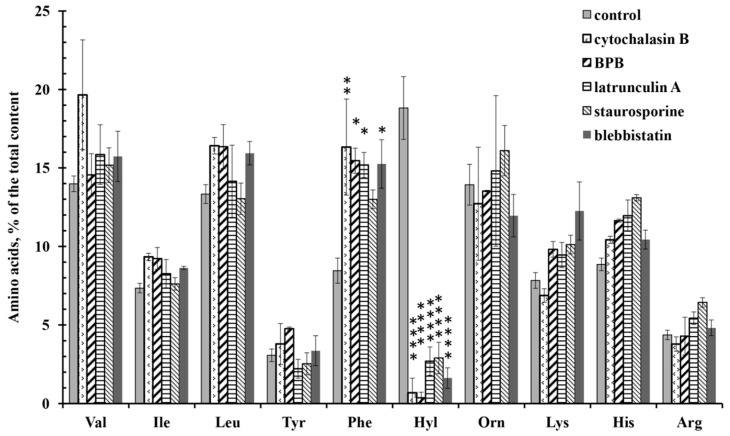
Free amino acid composition of neutrophil secretion during adhesion to fibronectin in the presence of drugs affecting the actin cytoskeleton. Human neutrophils were incubated over fibronectin-coated substrata for 20 min or in the presence of: 10 µg/mL cytochalasin D; 25 µM 4-bromophenacyl bromide; 1 µM latrunculin A; 0.2 µM staurosporine (A); 10 µM blebbistatin. Amino acid profiles were obtained by summing the results of three independent experiments. The amount of amino acid is represented as a percentage of the total content of the detected free amino acids (mean ± SEM). *—significant differences when compared to the value for the same amino acid in the control cells (*—*p* < 0.01; **—*p* < 0.001; ****—*p* < 0.0001). (Val—valine; Ile—isoleucine; Leu—leucine; Tyr—tyrosine; Phe—phenylalanine; Hyl—hydroxylysine; Orn—ornithine; Lys—lysine; His—histidine; Arg—arginine).

Amino acid analysis showed that all tested drugs significantly inhibit the release of hydroxylysine by neutrophils during adhesion. The characteristic effect of drugs acting on the actin cytoskeleton was also a statistically significant increase in the secretion of phenylalanine (Figure 7). The release of other free amino acids did not differ significantly from the control data.

**Figure 8:**
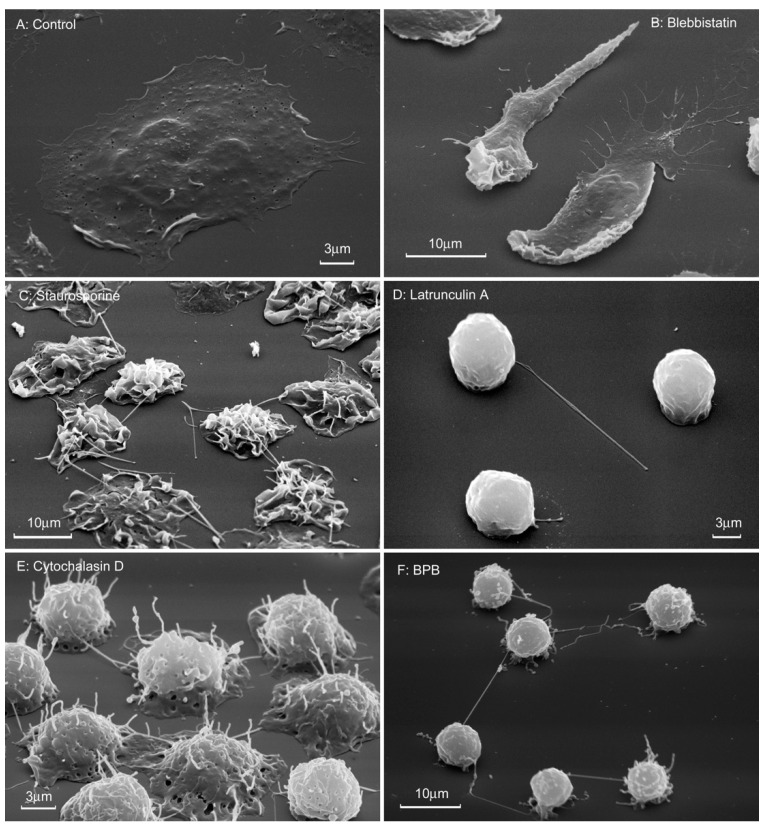
Morphology of neutrophils that were attached to fibronectin in the presence of drugs that violate the integrity of the actin cytoskeleton. Scanning electron microscopy of human neutrophils attached to fibronectin-coated substrates for 20 min under control conditions (**A**) or in the presence of 10 µM blebbistatin (**B**), 0.2 µM staurosporine (**C**), 1 µM latrunculin A (**D**), 10 µg/mL cytochalasin D (**E**), or 25 μM 4-bromophenacyl bromide (**F**). Pictures represent typical images observed in three independent experiments.

Scanning electron microscopy showed that neutrophils, which were attached to fibronectin in the presence of drugs that disrupt the integrity of the actin cytoskeleton, flatten poorly on the substrate and have a predominantly spherical shape (Figure 8). Cytochalasin D, latrunculin A and 4-bromophenacyl bromide induce a uniform and strong suppression of neutrophil spreading, as determined by measuring the cell area on a fibronectin-coated surface (Table 1). In the presence of all these substances, with the exception of the blebistatin, tubulovesicular filamentous membrane structures or cytonemes (membrane tethers, tentacles) are formed on the surface of neutrophils (Figure 8C–F). Cytonemes appear to be projections of a modified secretory stream of neutrophils, which usually occur as secretory vesicles budding from the plasma membrane. Depolymerization of actin blocks the separation of membrane vesicles from the plasmalemma and from each other. As a result, the secretory process leaves the cell in the form of filamentous cytonemes [51,52,53,54,55].

### 3.5. Influence of Minoxidil and Doxycycline on the Composition of Protein Secretion by Neutrophils during Adhesion to Fibronectin

**Figure 9:**
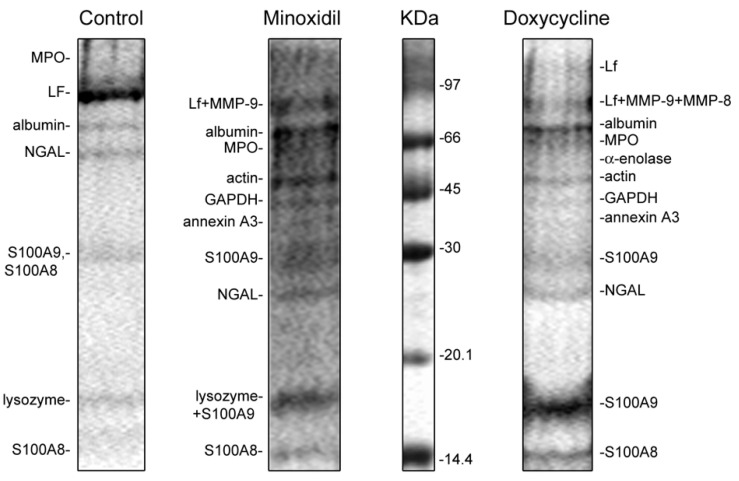
SDS-PAGE separation of proteins secreted by neutrophils in adherence to fibronectin in the presence of minoxidil and doxycycline. Human neutrophils were incubated over fibronectin-coated substrates for 20 min under control conditions or in the presence of 200 µM minoxidil or 20 µM doxycycline. Samples of extracellular medium were collected and proteins were extracted and subjected to 15% SDS-PAGE. Pictures represent typical protein profiles observed in the three independent experiments.

We also compared the effect of minoxidil and doxycycline on the composition of protein secretion by neutrophils during adhesion. Proteins were identified by mass spectrometric analysis after concentration and separation of proteins using electrophoresis. Silver staining of proteins in electrophoretic gels showed that neutrophils secreted multiple proteins during incubation (data not shown). However, Coomassie brilliant blue staining, which allows the identification of secreted proteins by mass spectrometry, is less sensitive (Figure 9). Not all, but the main secreted proteins were stained with Coomassie brilliant blue. These proteins form a stable protein profile of neutrophil secretion, which is characteristic for each investigated inhibitor under the same experimental conditions, but does not depend on donors [52,56].

The protein profiles of the secretion of neutrophils that adhere to fibronectin-coated substrates under control conditions included: secretory vesicle component albumin; secretory granule components lactoferrin (LF) and neutrophil gelatinase-associated lipocalin (NGAL); primary granule components lysozyme and myeloperoxidase (MPO); cytosolic S100A8 and S100A9 proteins. The proteins were located in the gel in accordance with their molecular weight with the exception of the 25 kDa protein, NGAL, which appeared as a 50 kDa protein. This can be explained by the ability of NGAL to form homodimers. The protein secretion composition of neutrophils attached to fibronectin under control conditions in this work (Figure 9, Control) corresponded to the previously published characteristic protein secretion profile of control neutrophils [52].

When neutrophils in suspension are exposed to stimuli such as fMLP or PMA, they first release the contents of the secretory vesicles, then the tertiary granules, then the secondary granules, and finally the primary granules [26]. The release of the granule content during cell adhesion occurs in a different order. In particular, adhesion stimulates the release of MPO, LF and NGAL, which are components of the primary and secondary granules [52,57]. The release of MMP-9, a tertiary granular component, occurred when neutrophils attached to fibronectin in the presence of fMLP, PMA or LPS [56].

In the present work, we observed that, in addition to proteins secreted by control cells, the protein profile of neutrophil secretion during adhesion in the presence of minoxidil or doxycycline included MMP-9 and MMP-8, as well as a number of cytosolic proteins such as actin and glycolytic enzymes α-enolase, glyceraldehyde-3-phosphate dehydrogenase (GAPDH) and annexin 3 (Figure 9, Table 2). In contrast to the secretion of control cells, NGAL was disposed in the gel according to a molecular weight of 25 kDa. The composition of the secretion of proteins by neutrophils attaching to fibronectin in the presence of minoxidil and doxycycline was basically the same.

## 4. Discussion

We have previously found that neutrophils secrete branched chain (valine, isoleucine, leucine), aromatic (tyrosine, phenylalanine) and positively charged free amino acids (arginine, ornithine, lysine, hydroxylysine, histidine) when incubated over an adhesive (fibronectin) or non-adhesive substrate at the same conditions. The difference was only in the content of hydroxylysine. The percentage of hydroxylysine in the secretion of neutrophils that attached and spread along fibronectin exceeded 20% of the total number of amino acids, but in the secretion of neutrophils that were incubated on a non-adhesive substrate did not exceed 2% [4]. The attachment and spreading of neutrophils on fibronectin selectively stimulated the release of hydroxylysine but did not notably affect the release of other free amino acids. The secretion of hydroxylysine was increased in the presence of insulin, but not glucagon or 17β-estradiol. There was no direct link between hydroxylysine release and cell activation with LPS or peptide fMLP, commonly used as activators of bactericidal functions of neutrophils. LPS stimulated, while fMLP strongly inhibited, the hydroxylysine release during neutrophil adhesion [4].

The increase in hydroxylysine release during neutrophil adhesion and spreading may be due to LH activation. The present work has demonstrated that minoxidil, an LH inhibitor, dramatically and selectively reduces adhesion-induced release of hydroxylysine (Figure 1), which coincides with inhibition of cell spreading (Figure 3; Table 1). Doxycycline, an inhibitor of MMP-9, also had a potent and selective inhibitory effect on the release of hydroxylysine (Figure 1), that coincided with significant inhibition of cell spreading (Figure 3, Table 1). The similar effects of minoxidil and doxycycline on neutrophil adhesion and secretion of hydroxylysine indicate a special interaction between LH and MMP-9 in these processes. No such interaction was observed for other neutrophil activity-ROS production: minoxidil stimulated ROS production, while doxycycline did not affect this process (Figure 4).

The mechanism of interaction between LH and MMP-9 is unknown. Both enzymes can enter the extracellular medium as a result of cell secretion and partially attach to the cell surface. Secretion of MMP-9 is low in resting cells, but increases significantly during tissue remodeling following wound healing or tumor invasion, and is thought to promote metastasis [58,59,60]. The attachment of MMP-9 to the cell surface via CD44 or other proteins can promote degradation of the extracellular matrix, protect the enzyme from natural inhibitors and promote cell invasion [22]. Recent data indicate that LH3 is a novel MMP-9 docking receptor on the cell surface of fibroblasts in the tumor stroma [21]. There are three LH 1–3 encoded in the human genome [9,10]. LH1 and LH2 are localized intracellularly in the ER in all studied tissues. LH3 is found in the ER, in the extracellular space and on the cell surface [8,61]. LH3 exhibits galactosyltransferase and glucosyltransferase activities along with LH activity. The site of lysyl hydroxylase activity ensures the retention of LH3 in the ER, while the site of glucosyltransferase activity is required for external secretion of LH3. [8,61]. Based on the literature data, it can be assumed that hydroxylysine is formed due to the interaction of MMP-9 with LH on the surface of neutrophils.

Minoxidil and doxycycline, in addition to suppressing the enzymatic activity of LH and MMP-9, can affect the formation of hydroxylysine by altering the secretion of enzymes (Figure 9, Table 2), as well as their association with each other and with the surface of neutrophils. Minoxidil and doxycycline in our experiments significantly changed the process of neutrophil secretion. MMP-9 was not detected in the extracellular environment of control neutrophils [52]. However, MMP-9 and, in addition, a number of cytoplasmic proteins, have been identified as the main proteins secreted by neutrophils treated with minoxidil or doxycycline (Figure 9, Table 2).

The close relationship between the processes of neutrophil adhesion and hydroxylysine secretion was confirmed by experiments with inhibitors of the PI3K/Akt pathway. In the presence of the inhibitors wortmannin and Akt 1/2, the release of hydroxylysine sharply decreased (Figure 5), and at the same time, the area of neutrophils was greatly reduced (Figure 6, Table 1). A close interaction between the LH and PI3K/Akt pathways also occurs in tumor cells. Knockdown of PLOD2 in glioma inactivates PI3K/Akt signaling, resulting in inhibition of invasion and metastatic formation [16].

Finally, all drugs that disrupt the integrity of the actin cytoskeleton almost completely suppressed the release of hydroxylysine during adhesion (Figure 7) and simultaneously sharply blocked the spreading of neutrophils on fibronectin (Figure 8, Table 1), demonstrating a close interaction between neutrophil adhesion and hydroxylysine release. We hypothesize that disruption of the actin cytoskeleton can inhibit the release of hydroxylysine due to a strong reorganization of neutrophil secretion [52,53,56]. Cytochalasin D has been shown to block the expression of CD44, the major MMP-9 docking site on the surface of various tumor cells and primary keratinocytes [25]. We hypothesize that depolymerization of the actin cytoskeleton may also alter the exposure of LH3, another molecule that can anchor MMP-9, to cells [21]. Various secretory pathways lead LH3 to the cell surface and to the extracellular space. LH3 molecules found in the extracellular environment are secreted through the Golgi complex. LH found on the cell surface bypasses the Golgi complex [8]. Changes in the functioning of the actin cytoskeleton can disrupt the balance between these secretory processes.

The results of our work do not answer many important questions. Where is hydroxylysine formed—intracellularly followed by secretion, in the extracellular environment or on the cell surface? Hydroxylysine can also be released as a result of the degradation of extracellular matrix proteins such as collagen. Urinary excretion of hydroxylysine and its glycosides is considered and used as an indicator of collagen degradation [62,63]. The fibronectin used in our experiments also contains collagen-like fragments. However, it would be difficult to explain the selective nature of the release of hydroxylysine upon adhesion as a result of protein degradation. The role of hydroxylysine and the multifunctional enzyme lysyl hydroxylase in the adhesion and other physiological functions of neutrophils is to be clarified in further studies.

Our data have shown a close relationship between neutrophil adhesion to fibronectin and hydroxylysine release. We have demonstrated that the lysyl hydroxylase inhibitor minoxidil, the matrix metalloproteinase inhibitor doxycycline, the PI3K/Akt pathway inhibitors wortmannin and the Akt1/2 inhibitor, as well as agents that disrupt the integrity of the actin cytoskeleton, selectively blocked both hydroxylysine release and neutrophil adhesion. Analysis of the literature shows that increased activity of LH, metalloproteinases and the PI3K/Akt pathway characterizes a number of tumors with increased invasiveness and the ability to form metastases. We assume that the formation of hydroxylysine is mediated by lysyl hydroxylase, which is activated upon adhesion of neutrophils in interaction with matrix metalloproteinase and the PI3K/Akt pathway when the actin cytoskeleton is intact. Drawing on an analogy with cancer cells, we hypothesize that these processes play important roles in the remodeling of the extracellular matrix, which is necessary for the invasion and adhesion of neutrophils to tissues.

## Figures and Tables

**Table 1:** Area of neutrophils attached to fibronectin in the presence of the inhibitors being tested. Data presented are mean ± SEM derived from cell area measurements on 15–20 neutrophils obtained with scanning electron microscopy in three similar experiments for each inhibitor. *—*p* < 0.0001 as compared to the control value. The cell area of the attached neutrophils was quantified using ImageJ image processing.

Treatment	Cell Area, µm^2^	% of Control
control	232 ± 9	100
Minoxidil, 200 µM	114 ± 25 *	49
Doxycycline, 20 µM	51 ± 3 *	22
cytochalasin D, 10 µg/mL	40 ± 5 *	17
4-bromophenacyl bromide, 25 µM	42 ± 2 *	18
latrunculin A, 1 µM	44 ± 1 *	19
Staurosporine, 0.2 µM	142 ± 9 *	61
Blebbistatin, 10 µM	109 ± 8 *	47
Wortmannin, 1 µM	85 ± 8 *	37
Akt ½ inhibitor, 25 µM	69 ± 4 *	30

**Table 2:** List of proteins secreted by neutrophils in adherence to fibronectin in the presence of minoxidil and doxycycline. Neutrophils were incubated over fibronectin-coated substrata for 20 min under control conditions (marked +), or in the presence of 200 µM minoxidil or 20 µM doxycycline. Proteins were separated by SDS-PAGE and identified by mass spectrometric analysis. Mass spectrometric analysis data were taken from experiments with doxycycline. Data for lysozyme were taken from experiments with minoxidil. Analogous proteins that were identified in control experiments are marked (+). The list includes proteins that have been reliably identified in three similar experiments.

Entres ID	Protein Name			Peptides Matched/Total	Coverage, %	MOWSE Score
	control.	minoxidil	doxycycline			
*Granular proteins*						
MMP9_HUMAN		MMP-9	MMP-9	22/58	35	137
MMP8_HUMAN			MMP-8	10/45	23	103
PERM_HUMAN	+	MPO	MPO	16/48	23	83
TRFL_HUMAN	+	LF	LF	17/58	36	153
ALBU_HUMAN	+	albumin	albumin	22/34	36	149
NGAL_HUMAN	+	NGAL	NGAL	11/18	55	156
LYSC_HUMAN	+	lysozyme		8/21	47	88
*Glycolytic enzymes*						
ENOA_HUMAN			α-enolase	10/24	30	72
G3P_HUMAN		GAPDH	GAPDH	12/27	57	89
*Cytoplasmic proteins*						
AKTB_HUMAN		actin	actin	12/26	36	127
ANXA3_HUMAN		annexin 3	annexin 3	7/20	27	64
S10A9_HUMAN	+	S100-A9	S100-A9	10/27	81	94
S10A8_HUMAN	+	S100-A8	S100-A8	10/11	53	156
